# Medicinal Mushrooms

**DOI:** 10.1155/2014/806180

**Published:** 2014-06-24

**Authors:** Ulrike Lindequist, Ha Won Kim, Evelin Tiralongo, Leo Van Griensven

**Affiliations:** ^1^Institute of Pharmacy, Ernst Moritz Arndt University of Greifswald, 17487 Greifswald, Germany; ^2^Department of Life Sciences, University of Seoul, Seoul 130-743, Republic of Korea; ^3^School of Pharmacy, Griffith University, Gold Coast Campus, QLD 4222, Australia; ^4^Plant Research International, Department of Bioscience, Wageningen University, 6700AA Wageningen, The Netherlands

Since beginning of mankind nature is the most important source of medicines. Bioactive compounds produced by living organisms can be used directly as drugs or as lead compounds for drug development. Besides, the natural material can be used as crude drug for preparation of powder or extracts.

Plants have traditionally been used as a source of most medical systems and as such herbal medicines constitute an important part of traditional and evidence-based medicine worldwide. In contrast though, the broad medicinal use of mushrooms was for a very long time restricted to Asian countries. Nowadays, the medicinal use of mushrooms, so called, medicinal mushrooms', is increasing also in Western and other countries. This is underlined not only by growing sales of mushroom products but also by an increasing number of scientific papers and international conferences in this field.

Medicinal mushrooms can be defined as macroscopic fungi, mostly higher Basidiomycetes, which are used in the form of extracts or powder for prevention, alleviation, or healing of diseases and/or for nutritional reasons.

Presently medicinal mushrooms are mainly used as dietary supplements or functional food. Nevertheless they have the potential to become real drugs of traditional and/or evidence-based medicine. To explore this potential high quality products, preclinical and clinical trials according to the regulations, and legal authorization are necessary.

The most important mushroom species are* Ganoderma lucidum*,* Coriolus versicolor* (L.:Fr.)Quél. (syn.* Trametes versicolor* [L.:Fr.]Pilát),* Lentinula edodes* (Berk.)Pegler,* Agaricus brasiliensis* Wasser et al.,* Cordyceps sinensis* (Berk.)Sacc. (*Ophiocordyceps sinensis* (Berk.)Sung & al.),* Grifola frondosa* (Dicks.:Fr.)Gray,* Hericium erinaceus* (Bull.:Fr.)Pers. and some others. Due to the extended traditional use of these mushrooms extensive knowledge about in vitro activities and mode of action and effects in animal assays is available. Some information about several chemical constituents responsible for the pharmacological effects is also available. Needless to say more research on the pharmacology and chemistry of these and others, so far less explored mushrooms, is urgently needed.

What's also missing, however, are investigations about structure-activity-relationships and possible toxicological risks of these mushrooms and their products, clinical trials and suitable quality critera for mushroom products and established methods for its control.

We received 13 research papers in this field which indicates that mushroom research is still very limited. However, this special issue includes 6 high-quality peer-reviewed papers demonstrating essential new findings about different pharmacological effects of several medicinal mushrooms and their components in vitro, in animal assays and in humans.

Chan et al. and Kawai et al. showed that* Amauroderma rugosum *and* Pleurotus eryngii *have promising antiinflammatory properties.* A. rugosum *is used by indigenous communities in Malaysia,* P. eryngii *is consumed as fresh cultivated mushroom worldwide.* Clitocybe nuda*, also known as* Lepista nuda*, is an edible mushroom in Europe. It is known for its antioxidative and antimicrobial properties. The study by Shi et al. validated the antidiabetic and hypolipidemic effects of* Clitocybe nuda *in diabetic mice and gives detailed insight into its mode of action.

Beta-glucans (MBGS) constitute one of the most important groups of bioactive compounds in mushrooms. They have been reported as anticancer agents mainly by strengthening immune activities. The papers of Chen et al. and Wu et al. focus on investigations of beta-glucans from* Ganoderma lucidum*, the famous Reishi or Ling Zhi mushroom. The beta-glucans isolated from solid culture of* Ganoderma lucidum *inhibit, in combination with radiation, tumor metastasis in Lewis lung carcinoma bearing mice (Chen at el.), whereas the oral administration of MBG, also obtained from mycelium of* G. lucidum*, modulates immune responses in an allergy murine model (Wu et al.).

The study of Rossi et al. investigated the influence of a mixture of* Ganoderma lucidum* and* Ophiocordyceps sinensis *on the performance and stress resistance of cyclists by monitoring the testosterone/cortisol ratio in saliva, as well as oxidative stress. Although only a small number of participants were included in this study, the presented results confirm the potential clinical use of mushrooms in general and the protection of the athletes from overtraining syndrome in particular.

